# Long-Term Nitrogen Amendment Alters the Diversity and Assemblage of Soil Bacterial Communities in Tallgrass Prairie

**DOI:** 10.1371/journal.pone.0067884

**Published:** 2013-06-28

**Authors:** Joseph D. Coolon, Kenneth L. Jones, Timothy C. Todd, John M. Blair, Michael A. Herman

**Affiliations:** 1 Kansas State University, Ecological Genomics Institute, Division of Biology, Manhattan, Kansas, United States of America; 2 University of Michigan, Department of Ecology and Evolutionary Biology, Ann Arbor, Michigan, United States of America; 3 University of Colorado School of Medicine, Department of Biochemistry and Molecular Genetics, Aurora, Colorado, United States of America; 4 Kansas State University, Department of Plant Pathology, Manhattan, Kansas, United States of America; Universidad de Salamanca, Spain

## Abstract

Anthropogenic changes are altering the environmental conditions and the biota of ecosystems worldwide. In many temperate grasslands, such as North American tallgrass prairie, these changes include alteration in historically important disturbance regimes (e.g., frequency of fires) and enhanced availability of potentially limiting nutrients, particularly nitrogen. Such anthropogenically-driven changes in the environment are known to elicit substantial changes in plant and consumer communities aboveground, but much less is known about their effects on soil microbial communities. Due to the high diversity of soil microbes and methodological challenges associated with assessing microbial community composition, relatively few studies have addressed specific taxonomic changes underlying microbial community-level responses to different fire regimes or nutrient amendments in tallgrass prairie. We used deep sequencing of the V3 region of the 16S rRNA gene to explore the effects of contrasting fire regimes and nutrient enrichment on soil bacterial communities in a long-term (20 yrs) experiment in native tallgrass prairie in the eastern Central Plains. We focused on responses to nutrient amendments coupled with two extreme fire regimes (annual prescribed spring burning and complete fire exclusion). The dominant bacterial phyla identified were Proteobacteria, Verrucomicrobia, Bacteriodetes, Acidobacteria, Firmicutes, and Actinobacteria and made up 80% of all taxa quantified. Chronic nitrogen enrichment significantly impacted bacterial community diversity and community structure varied according to nitrogen treatment, but not phosphorus enrichment or fire regime. We also found significant responses of individual bacterial groups including Nitrospira and Gammaproteobacteria to long-term nitrogen enrichment. Our results show that soil nitrogen enrichment can significantly alter bacterial community diversity, structure, and individual taxa abundance, which have important implications for both managed and natural grassland ecosystems.

## Introduction

Understanding biotic responses to anthropogenically-driven environmental changes is a central focus of many ecological studies, and is crucial for predicting the long-term consequences of contemporary and future human-induced changes in the Earth’s ecosystems. Determining the responses of microbial communities is particularly important as they are responsible for the majority of energy and nutrient transformations in most ecosystems [1], are the most basal components of soil and aquatic food webs and exert widespread effects on ecosystem function. Many recent studies in a broad range of ecosystems indicate that the composition of soil microbial communities is sensitive to a variety of land use changes [2]. For example, wholesale land use conversions, such as deforestation [3], [4] or tilling for agriculture [5], [6], cause shifts in the bacterial community resulting in decreased overall diversity and/or disruption of biogeochemical processes, leading to alterations in ecosystem functioning. Even seemingly less intrusive soil perturbations such as periodic fires in forests and woodlands [3], [4], [7] or nitrogen enrichment in a variety of ecosystems [8], [9], [10], [11], [12] can alter microbial communities (but see [13]).

In the tallgrass prairie, where the predominant land-use change historically was conversion of native grasslands to agriculture [14], disturbances such as tilling [15], [16], [17], burning [15], [18], fertilization and irrigation [17], [19] elicit changes in soil biogeochemical processes and properties. Previous research in tallgrass prairie has documented a myriad of above- and below-ground responses to these disturbances. However, there have been fewer studies of soil microbial community responses to disturbance, and changes in the soil microbial community are often inferred based on biogeochemical changes or changes in frequency of microbial biomarkers in the soil [15], [16], [18], [19]. Recently, researchers have turned to deep sequencing of microbial communities to assess changes in bacterial composition and diversity in response to perturbation in a variety of environmental contexts [10], [20], [21], [22].

Our objective was to determine how bacterial communities respond to two important contemporary environmental changes common to tallgrass prairie ecosystems in the eastern Central Plains: changes in fire frequency and nitrogen enrichment. Periodic fires were historically common in these grasslands and prescribed burning is a common management practice for maintaining grass dominance and suppressing the growth and cover of woody vegetation. Fire suppression in these grasslands can lead to rapid transitions to shrubland and woodland. While fire has direct negative effects on some plant species (e.g., eastern red cedar), prescribed burning often takes place in the dormant season (late winter or early spring) and the indirect effects of fire on the soil environment and subsequent plant resource use and competition are generally considered to more important than direct effects. Surface fires are generally fast moving and, in contrast to forest, woodland or tundra fires, there is little heat penetration in soils and little direct effect on soils properties or soil communities. Instead, the major effects of fire in these grasslands are due to removal of accumulated standing dead and surface plant litter (detritus), the loss of nitrogen through volatilization of nitrogen in detritus, and resulting changes in the post-fire soil environment. Removal of accumulated detritus results in more rapid warming in the spring and a more favorable light environment for emerging plants. These post-fire environmental changes are important in structuring plant communities [23], [24] and affecting soil biophysical properties and nutrient transformations [25], [26]. In general, fire in tallgrass prairie enhances above- and belowground plant productivity [27] and increases the dominance of a few species of perennial C_4_ (warm-season grasses) while reducing the cover of subdominant species and lowering overall plant species richness [23].

Nitrogen is generally considered to be a limiting nutrient in these grasslands, and N enrichment alters both plant productivity and community composition [28], [29]. Although experimental nitrogen enrichment increases plant productivity, it also leads to declines in species richness and diversity [30]. Levels of nitrogen enrichment in these studies often exceed current atmospheric N deposition rates, but even relatively low levels of chronic nitrogen enrichment are a threat to grassland conservation [31]. Further, interactions between changes in fire frequency and N enrichment are expected, since one of the effects of frequent fire is to reduce soil nitrogen availability [25] and to increase conservative recycling of nitrogen in the plant-soil system [18]. Here we use long-term experimental manipulations of fire regimes and nutrient enrichment in native tallgrass prairie to address three hypotheses; 1) soil bacterial diversity is altered in response to nutrient enrichment and/or contrasting fire regimes (annually burned vs. unburned treatments), 2) soil microbial community structure is altered in response to nutrient enrichment and/or contrasting fire regimes, 3) individual taxa frequencies are altered in response to nutrient enrichment and/or contrasting fire regimes. To do this we used deep sequencing targeting the V3 region of the bacterial 16S rRNA gene to identify and quantify soil bacterial taxa from replicated factorial field experiment at the Konza Prairie Long-Term Ecological Research (LTER) site.

## Materials and Methods

### Study Design, Soil Sampling and DNA Extraction

This research utilized an ongoing experiment established and supported by the Konza Prairie Long-Term Ecological research (LTER) program, and was approved by the Konza Prairie Biological Station, a 3487-hectare area of native tallgrass prairie in the Flint Hills of northeastern Kansas. Konza Prairie Biological Station is jointly owned by Kansas State University and The Nature Conservancy, and managed by the KSU Division of Biology. No protected species were sampled in association with this study. We sampled soil bacterial communities from the Belowground Plot Experiment, which was initiated in 1986 to assess above- and below-ground responses of native tallgrass prairie communities to contrasting fire regimes and nutrient addition treatments. The treatments used here were: burning (annually burned vs. unburned); ammonium nitrate addition (10 g N/m^2^ annually vs. no addition); and superphosphate addition (1 g P/m^2^ annually vs. no addition). The experimental design consisted of eight large plots (50 m×25 m) subdivided into a total of 32, 12.5 m×12.5 m subplots (Figure S1). Treatments were arranged in a split-strip plot design with four replications per treatment combination. Burning treatment was assigned at the whole plot level and fertilization (nitrogen and phosphorus) additions were imposed as strip-plot treatments. Burning was performed in mid-March to mid-April of each year (burn applied March 28 in 2006) and fertilizer treatments were applied in late May to early June.

For each of the 32 plots sampled, two 2.5 cm diameter, 10 cm deep cores were collected post fire on April 11, 2006, pooled, and homogenized. We chose to sample to 10 cm as previous studies have indicated significant effects of contrasting fire regimes and nutrient additions on belowground plant biomass and soil properties at this depth [26], [28]. From each composite sample, genomic DNA was extracted from a 10 g subsample using a standard soil DNA extraction kit (UltraClean Mega Soil DNA Kit, MoBio, Carlsbad, CA). After extraction, the DNA was diluted to ∼5 ng/µl and stored at −80°C.

### Soil Physico-chemical Characteristics

Selected soil characteristics were assessed by the Kansas State University Agronomy Soil Testing Lab from samples collected prior (samples collected in June 2004) to those used for microbial community analyses (Table 1). Briefly, soil for all measurements was prepared for all measurements by drying overnight at 50°C followed by passage through a 2-mm sieve. pH was measured directly using a 1∶1 slurry of d soil and deionized water. Concentrations of available soil phosphorus were determined by Bray phosphorus test. Exchangeable cations (Ca_2_^+^, Mg_2_^+^, K^+^, Na^+^) were measured by flame atomic absorption or ISC spectroscopy following extraction with 1 M ammonium acetate (pH 7). Extractable ammonium (NH_4_^+^) and nitrate (NO_3_^−^) were determined by colorimetric assay using an automated analyzer. Total nitrogen and phosphorus were measured colorimetrically following a modified Kjeldahl digestion. Organic matter content (%) was measured by the Walkley-Black method (Table 1). For more details see the Kansas State University Agronomy Soil Testing Lab (http://www.agronomy.ksu.edu/soiltesting/).

**Table 1:** Average soil properties in the Belowground Plot Experiment.

Treatment	pH	Bray P	Ca	K	Mg	Na	NH_4_−N	NO_3_−N	N_ext_	OM	Total N	Total P
Burn	6.26 (0.08)	4.25 (0.75)	3612.81 (153.96)	371.10 (18.58)	415.01 (36.90)	10.04 (0.88)	3.44 (0.30)	0.48 (0.28)	3.90 (0.45)	4.65 (0.40)	0.204 (0.012)	0.034(0.0009)
Burn+Nitrogen	5.94 (0.11)	2.75 (0.48)	3203.75 (101.39)	283.74 (7.33)	512.66 (17.10)	10.68 (1.45)	3.58 (0.34)	0.95 (0.35)	4.53 (0.48)	4.23 (0.14)	0.192 (0.008)	0.032(0.0007)
Control	6.21 (0.20)	4.25 (1.11)	3485.93 (277.16)	393.69 (79.93)	437.86 (69.08)	12.92 (2.39)	3.95 (0.53)	0.56 (0.21)	4.53 (0.57)	4.48 (0.20)	0.205 (0.008)	0.033(0.0017)
Nitrogen	5.92 (0.23)	6.00 (3.03)	3690.17 (145.59)	335.69 (27.03)	416.81 (53.20)	12.45 (3.20)	4.80 (0.65)	3.87 (1.20)	8.68 (1.80)	4.35 (0.38)	0.210 (0.02)	0.032 (0.0009)
p-values from general linear model:												
**Effect**	**pH**	**Bray P**	**Ca**	**K**	**Mg**	**Na**	**NH_4_−N**	**NO_3_−N**	**N_ext_**	**OM**	**Total N**	**Total P**
Burn	0.8504	0.3504	0.3419	0.4068	0.4626	0.305	0.0951	0.0382	0.0341	0.9352	0.4738	0.5641
Nitrogen	0.0933	0.9417	0.5833	0.1195	0.4414	0.9708	0.3175	0.0127	0.0341	0.3792	0.7939	0.1525
Burn X Nitrogen	0.9076	0.3504	0.1172	0.7407	0.2409	0.803	0.4706	0.0488	0.103	0.6275	0.5073	0.5996

Soil properties (and units) measured in the Belowground Plot Experiment included: pH, Bray Phosphorus (ppm), exchangeable Calcium (ppm), exchangeable Potassium (ppm), exchangeable Magnesium (ppm), exchangeable Sodium (ppm), NH_4_−Nitrogen (ppm), NO_3_−Nitrogen (ppm), extractable inorganic Nitrogen (ppm), Organic matter (%), total Nitrogen (% weight), and total Phosphorus (% weight). Treatment means and standard error is given for each parameter. P-values from general linear model testing effects listed are shown below.

### 16S rRNA Amplification for Deep Sequencing

DNA extracts of soil bacterial communities from each of the 32 plots sampled were amplified using universal bacterial primers (U341F and U533R, [32]) designed to amplify the third variable region (V3) of the 16S rRNA gene. The V3 region was chosen because preliminary analyses suggested that it had high primer site conservation and would maximize the information obtained using ∼100 bp sequences from soil samples. This was subsequently confirmed by Vaseleiadis *et al.* [33] using an *in silico* approach that identified it as one of the most variable stretches of the 16S gene and results suggested that for sequence frequencies similar to those found in soils, the V3 region had superior performance compared to other variable sites in the 16S gene [33]. These primer constructs (Table S1) also incorporated MPS sequencing primers [34] and a unique 5-bp sequence of DNA between the sequencing primer and the reverse 16S primer. These barcoded primer constructs were specifically designed to allow randomized sequencing from a mixture of multiple PCR products. PCR reactions (1x Amplitaq Gold polymerase reaction buffer (Applied Biosystems, Foster City, CA), 3.75 mM MgCl_2_, 200 µM dNTP, 0.5 µM each forward and reverse _b_MPS 16S primers, one unit Amplitaq Gold LD polymerase, and 5 ng soil extracted DNA) were run for 25 cycles at 95°C for 1 minute, 55°C for 1 minute, 74°C for 1 minute on an iCycler IQ real-time thermocycler (Bio-Rad Laboratories, Hercules, CA). PCR reactions were spiked with SYBR Green and run on a realtime thermocycler to assure that the PCR was stopped during log-phase. PCR amplification was done in triplicate, individual reactions pooled, and cleaned using an AMPure PCR cleanup kit (Agencourt Bioscience, Beverly, MA). We utilized a single MPS run with four blocks and randomly assigned 32 DNA samples to one of 32 different barcoded primers (Table S1) in one of the four sequencing blocks and amplified the V3 region of the 16S rRNA gene independently using the appropriate primers. For the sequencing block used for this study, 100 ng of each differentially barcoded PCR product were pooled and sequenced by 454 Life Sciences (Branford, CT; 2006) generating 71,769 sequences. Sequences generated for this study have been deposited in the NCBI Short Read Archive under accession SRP001373.

### Bioinformatics and OTU Designation

Following previously described methodologies [35], raw sequences were searched for the occurrence of the primer barcode immediately preceding the 16S primer sequence. Where found, the barcode was removed and the corresponding plot designation was incorporated into the sequence name. Sequences were removed if they did not contain a valid primer or barcode sequence, contained more than one ambiguous base, or were shorter than 85 bp (low quality sequences) or longer than 125 bp (containing long homopolymers), to reduce the number of sequence errors [36]. An average of 1,584 sequences per plot that were on average 101 bases long were retained for analyses (Table S2). For the remaining sequences, CAP3 [37] was used to align sequences at each of 34 sequence identity levels (SILs, 66% to 99%) using a minimal overlap of 75 bp to generate operational taxonomic units (OTUs).

### Rarefaction Curves

**Figure 1:**
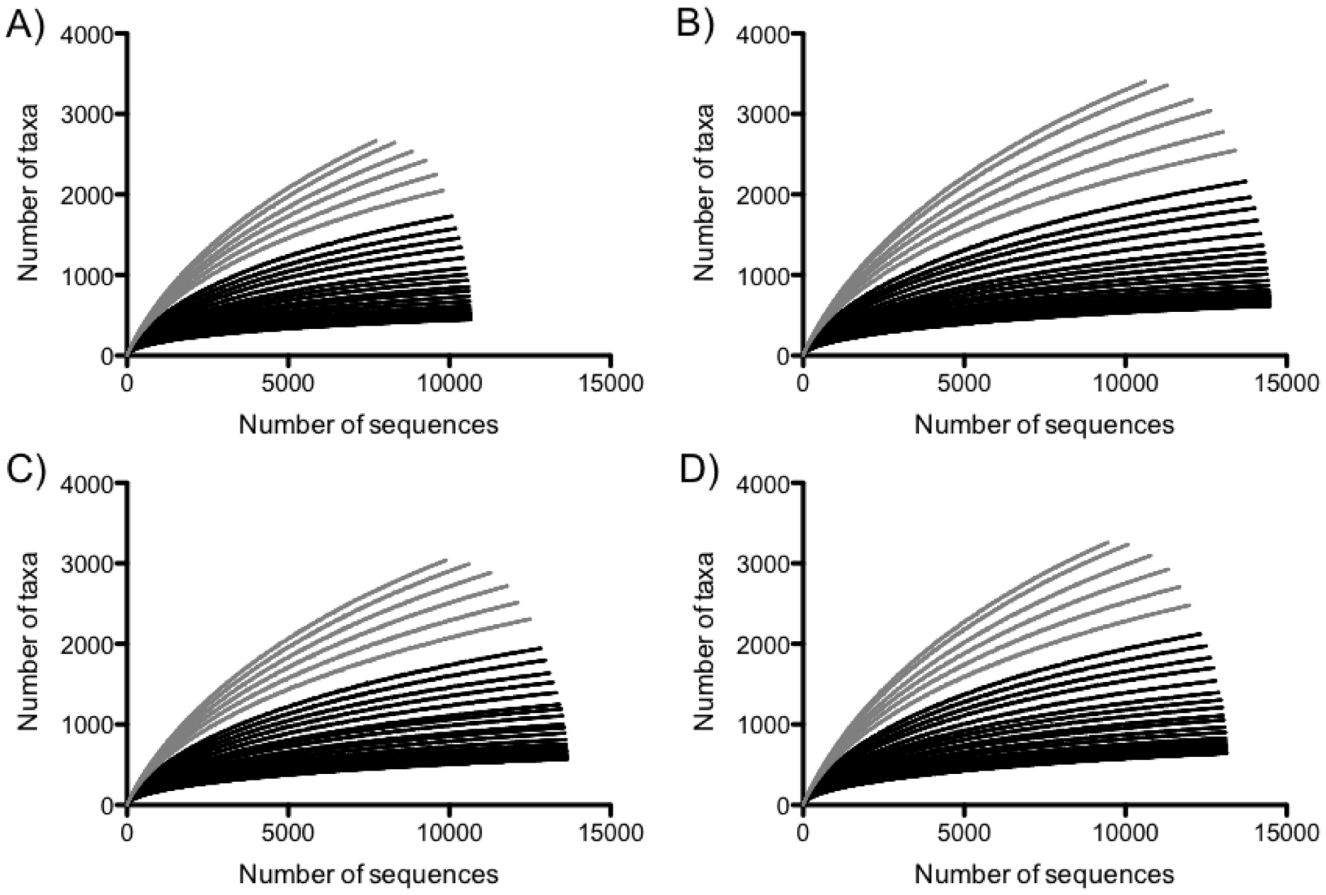
Rarefaction curves calculated independently for the cumulative treatments in the: A) burn+nitrogen, B) burn, C) nitrogen, and D) control. Black lines indicate curves for 66% SIL (bottom black line) to 93% SIL (top black line); gray lines indicate curves for 94% SIL (bottom gray line) to 99% SIL (top gray line).

We calculated OTU accumulation curves (i.e., rarefaction curves) at all percent sequence identity levels (%SILs) in the sequence sampling range (66% to 99% sequence identity) for the main treatment effects and CAP3 results were pooled for each treatment. Within treatments, the pooled collection of observed sequences were sampled at increasing intervals of 1 (e.g., 1 sequence, 2 sequences, etc.) and the number of OTUs identified within each subsample of sequences was determined. Rarefaction curves were produced from ten replicate samplings at each of the sampling intervals and means reported for each interval for the pooled collection of sequences (Figure 1). In addition, in order to better evaluate the beta-diversity calculations we generated rarefaction curves for each plot at 93 and 97% SIL (Figure S2).

### Diversity Indices

Overall taxonomic richness (S) was calculated by summing the number of OTUs at 93% SIL or 97% SIL, including singlets, which occurred within each plot. Simpson’s Diversity (∑p_i_^2^) and Shannon’s Diversity (*e*∑p_i_(ln(p_i_))) were calculated for each plot, where p_i_ is the frequency of occurrence of each OTU. Evenness was calculated as the ratio of Shannon’s Diversity and richness (*e*∑p_i_(ln(p_i_)/S). A final index of diversity, Fisher’s alpha log-series [38] was calculated iteratively using the equation S/N = ((1−x)/x)(−ln(1−x)), where S is richness and N is the total number of sequences within the plot. Once x was solved, the diversity index alpha (α) was calculated as N(1−x)/x. Differences in diversity across treatments were analyzed using the MIXED procedure in SAS (SAS Institute Inc., Cary, NC).

### OTU Frequency

Our rarefaction analysis indicated that 93% SIL was the most exclusive sufficiently sampled %SIL (Figure 1). However, since 97% SIL is commonly used in many bacterial community studies, we used both 93% and 97% SIL for OTU frequency analyses. For taxa at 93% or 97% SIL whose abundance was significantly different from zero (determined using a T-test in SAS; SAS Institute Inc., Cary, NC), a mixed model ANOVA was preformed to determine treatment effects, using the MIXED procedure in SAS. This procedure was included to reduce inclusion of erroneous rare taxa and increase the accuracy of taxa abundance measures. Phylum level taxa were also analyzed using the same model. Due to the multitude of simultaneously conducted tests, we used a false discovery rate (FDR) correction to account for the increase in type 2 error using the q-value package in R version 2.8.1 [39]. Those OTUs identified as having significant responses to treatments were identified with the naïve Bayesian classifier tool [40] in the Ribosomal Database Project (RDP 10.26, http://rdp.cme.msu.edu/, [41], [42]) using a bootstrap cutoff of 50% as recommended for sequences in the length range generated for this study. Due to lengths of sequences generated for this study, we were not able to obtain genus-level taxonomic names for all taxa identified as significantly responsive to treatments. Therefore, we reported the taxonomic designation that was best supported by the naïve Bayesian classifier tool in the Ribosomal Database Project.

**Figure 2:**
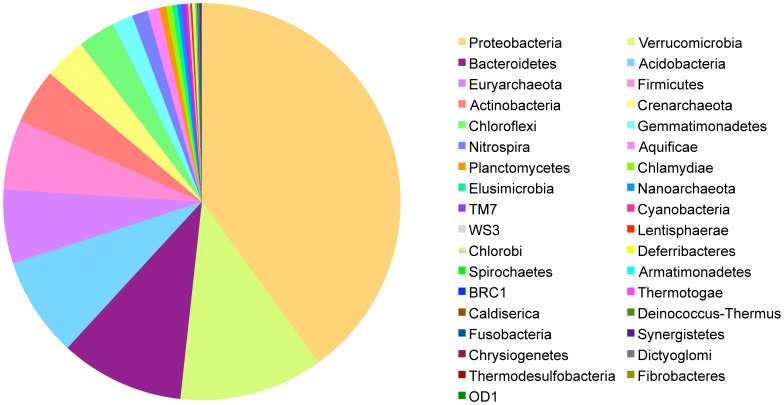
Bacterial phylum identified in soil samples. Bacterial frequency is shown for all plots sampled. Frequencies for each phylum in each treatment are show in Table S2 and plotted in Figure S3.

Fast UniFrac [43], [44] was used to evaluate the relatedness of the bacterial communities within soil samples. We clustered sequences at 93% and 97% SIL using CAP3 and used one randomly selected sequence from each OTU as a representative. Sequences were aligned with MUSCLE [45] using the default parameters generating the input tree for UniFrac. Normalized Principle Coordinate Analyses (PCoA), weighted by OTU abundances, were performed to evaluate similarity between samples. The representative sequences from each OTU at 93% SIL used in the UniFrac analyses were also used to determine taxonomy with the naïve Bayesian classifier tool [40] in the Ribosomal Database Project and abundances of different groups were determined at the phylum level (Figure 2).

## Results

### Rarefaction Curves of Bar-coded Bacterial 16S rRNA Sequences

To quantify the bacterial community we used bar-coded 16S rRNA 454 sequencing. After removing aberrant, short and/or long sequences, a total of 47,508 sequences were retained for further analyses (Table S2). These quality-filtered sequences were assigned operational taxonomic units (OTUs) at each of 34% SIL (66% to 99%). At each level of sequence identity, sequences were parsed by plot and used to calculate the frequency of occurrence of all OTUs for each of the 32 plots sampled. To assess the quality of the data and determine where sampling of the microbial community was sufficient, we calculated rarefaction curves for the cumulative plots within each treatment. Figure 1 suggests that OTUs generated at 66–93% sequence identity (black lines in Figure 1) reached saturation indicating appropriate sampling intensity at lower sequence identity levels, while those generated at 94–99% sequence identity (gray lines in Figure 1) did not reach saturation. Thus our rarefaction analyses indicated that 93% SIL was the most taxonomically exclusive level for which a sufficient level of sampling was achieved (Figure 1). In addition, because 97% SIL is commonly used in the literature to determine and analyze OTU frequencies, we performed subsequent diversity analyses at both 93% and 97% SIL. In order to better evaluate the beta-diversity calculations we generated plot-level rarefaction curves for 93% and 97% SIL (Figure S2) and observed that the patterns were similar to treatment-level rarefaction curves (Figures 1, S2).

### Bacterial Community Diversity in Different Treatments

To determine whether soil bacterial diversity was significantly altered in response to nutrient enrichment and contrasting fire regimes, we first determined the bacterial taxa present in the soils sampled. To do this we used OTU abundances and grouped the OTUs at the level of phylum (Table S3). Interestingly, we found that around 10% of the sequences generated were derived from Archaea, which is greater than previously reported values for grassland soils which are typically around 5% Archaea [46]. However this is consistent with reports of relatively high and variable Archaeal abundance in terrestrial ecosystems in general, and an increasing awareness of their potential roles in nutrient transformations in surface soils [46]. Proteobacteria were the most abundant (40%) of the bacterial phyla identified in the tallgrass prairie soils sampled, followed by Verrucomicrobia (11.7%), Bacteriodetes (10.1%), Acidobacteria (8.1%), Firmicutes (5.7%) and Actinobacteria (4.5%) with the remaining bacterial and Archaeal phylum summing to 20% (Figures 2, S3).

**Figure 3:**
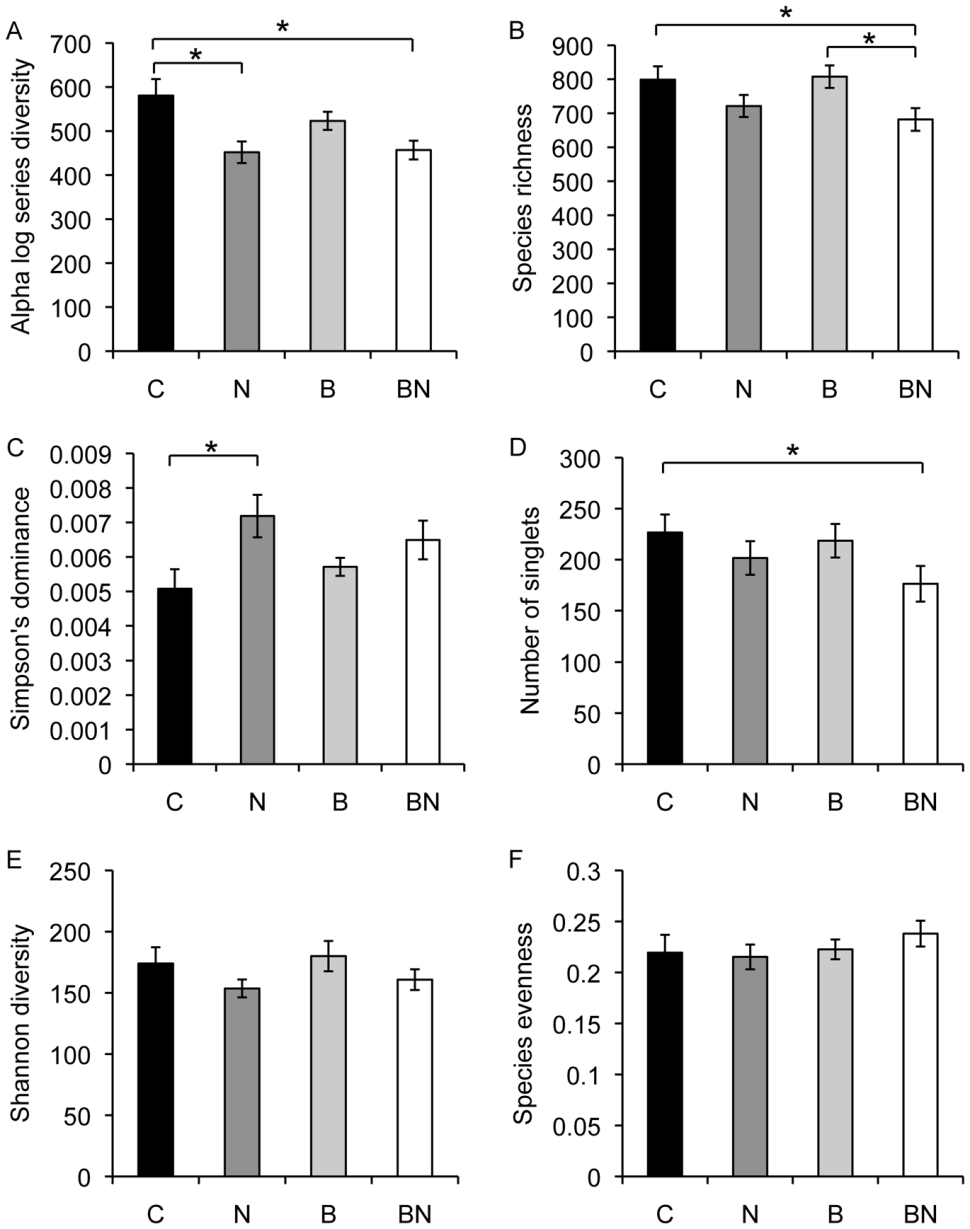
Mean alpha diversity for control (black), nitrogen amended (dark gray), burned (light gray), and burn+nitrogen treatment (white). A) Fisher’s alpha, B) Taxonomic richness, C) Simpson’s dominance, D) number of singlets (extremely rare taxa), E) Shannon diversity and F) taxonomic evenness are shown. A * indicates significant at p<0.05 in mixed model ANOVA. Error bars indicate standard errors.

To determine treatment effects on the bacterial community, OTU frequencies generated at 93% and 97% SIL were used to calculate taxonomic richness, number of extremely rare or singlet taxa, dominance, diversity, and evenness for each plot and analyzed across treatments using ANOVA. Significant changes in alpha diversity were observed (Figures 3 and S4). The main effects of burning and phosphorus amendment (not shown) were not significant for any of the diversity indices at either %SIL (p>0.05 for all tests). The nitrogen treatment significantly reduced taxonomic richness of OTUs generated at 93% SIL (p = 0.008) but not the OTUs generated at 97% SIL. However nitrogen treatment did significantly reduced Fisher’s diversity for OTUs generated at 93% and 97% SIL (p = 0.001 and 0.007, respectively) and significantly reduced the number of extremely rare or singlet taxa at 93% SIL (p = 0.05), but not at 97% SIL. There was also a significant increase in Simpson’s dominance in response to nitrogen addition at both SILs (p = 0.009 and 0.02). The nitrogen addition treatment, however, did not significantly change Shannon diversity or taxonomic evenness (Figures 3, S4). In specific contrasts of the treatment combinations, we found significant differences between control and nitrogen treatments at both %SIL (p = 0.002 and 0.013) and between control and burn+nitrogen treatments (p = 0.004 and 0.02) for Fisher’s diversity. We also found significant differences between control and burn+nitrogen treatments at 93% SIL (p = 0.031); and between burn and burn+nitrogen treatments at both %SIL (p = 0.018 and 0.04) for species richness, between control and nitrogen treatments at both %SIL (p = 0.007 and 0.005) for Simpson’s dominance, and between control and burn+nitrogen treatments at 93% SIL (p = 0.046) in the number of singlet taxa (Figure 3). Thus, while there were some differences the analyses at 93% and 97% SIL were highly similar, indicating that our diversity results were robust to the %SIL level at which OTUs were determined.

### Phylogenetically Informed Bacterial Community Analysis

**Figure 4:**
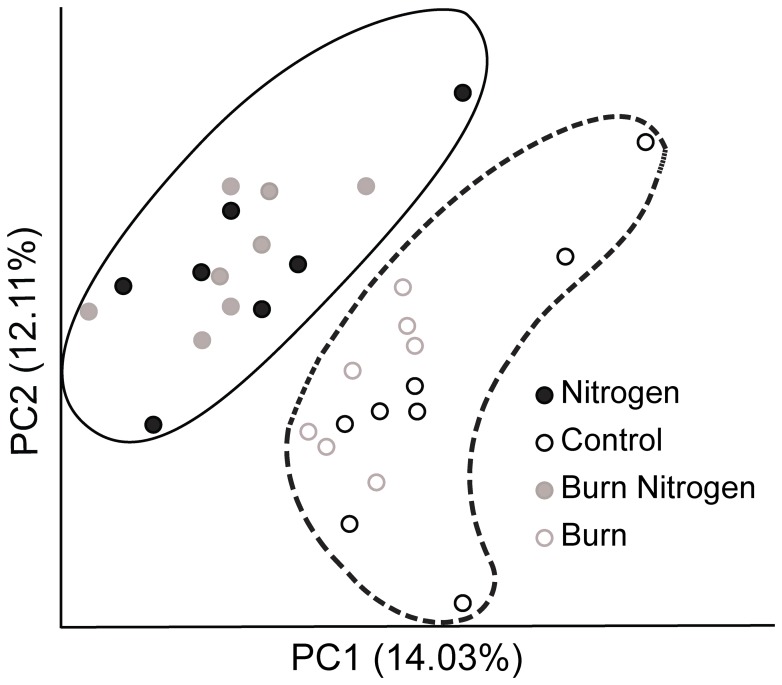
UniFrac Principle Coordinate Analysis plots for the different experiments in this study. Treatments indicated are control (open black), nitrogen addition (filled black), burned (open grey), and nitrogen+burn (filled grey) treatments were plotted. Clustering based on nitrogen treatment is outlined (nitrogen in solid line and no nitrogen addition in dashed line).

To determine whether soil microbial community structure was altered by nutrient enrichment and/or contrasting fire regimes, we used the UniFrac metric [43], [44] with Principle Coordinates Analysis (PCoA) to determine the relatedness of bacterial communities between pairs of samples. The UniFrac metric uses the overlap in branch lengths to estimate the phylogenetic distance, or relatedness, between pairs of bacterial communities [47] and the PCoA clusters related communities. Comparison of soil microbial community makeup using UniFrac PCoA for OTUs generated at 93% or 97% SIL showed distinct clustering by nitrogen treatment (Figures 4, S5), but there was no detectable clustering by burn treatment. Nitrogen amended plots were separated from non-nitrogen amended plots on the first two PCoA axes, which together represented 26% or 10% of the total variance for 93% or 97% SIL, respectively.

To better understand how the treatments could indirectly elicit changes in bacterial community structure we investigated how the different treatments altered soil chemical properties (Table 1). As expected, long-term nitrogen fertilization significantly increased the concentrations of extractable inorganic nitrogen in the soil (p = 0.0341), due primarily to a significant increase in nitrate concentration (p = 0.0127). We also found that burning significantly reduced extractable soil inorganic nitrogen concentrations (p = 0.0382) and we observed a significant interaction of burn and nitrogen treatments on nitrate concentrations (p = 0.0488). The increases in total extractable inorganic nitrogen and nitrate concentrations in response to nitrogen amendment alone were completely mitigated by the concurrent application of the annual burning treatment and associated N losses [25], making levels of extractable inorganic nitrogen in the control and burn+nitrogen treatments indistinguishable (Table 1). No other measured soil property differed by treatment.

### Frequencies of Individual Bacterial Taxa in Different Treatments

Since our analyses indicated that nitrogen addition altered microbial diversity (Figure 3, Figure S4), we examined how individual taxa responded to these perturbations. To test the hypothesis that individual taxon frequencies were altered in response to nutrient enrichment and/or contrasting fire regimes we performed a mixed model ANOVA on OTU frequency of occurrence at the phylum level, 93% SIL and 97% SIL. Interestingly, we found that phylum frequencies were quite similar across treatments and there were no significant treatment effects on phylum-level bacterial taxa frequencies (Figure S3). However, when we examined taxa at 93% SIL, we found that multiple taxa responded significantly to nitrogen amendment and to the interaction of nitrogen amendment and burning treatments, with most taxa showing significant response to treatment interactions (Table 2). We found that OTUs identified as Burkholderiales and Acidobacteria Gp1 responded significantly to nitrogen addition only, while all other taxa that exhibited a significant main effect response to nitrogen also exhibited a significant interactive response to nitrogen addition and burn treatments (Table 2). The Burkholderiales OTU was significantly reduced while the Acidobacteria Gp1 OTU significantly increased in response to nitrogen amendment (Table 2). OTUs corresponding to Nitrospira and Gammaproteobacteria were increased in response to nitrogen addition, with significant differences observed between burn and burn+nitrogen treatments. Similarly, an Acidobacteria Gp3 OTU increased in frequency in response to nitrogen addition, with significant differences observed between control and nitrogen amended plots in the fire exclusion treatment. An OTU classified as Acidobacteria Gp4 exhibited a significant interaction between nitrogen addition and burn treatments, with a decrease in response to nitrogen addition only in the presence of burning. An OTU classified as Ohtaekwangia and an OTU classified as Flavobacteriaceae showed more complex significant interactions between burn and nitrogen addition treatments and an Alphaproteobacteria OTU had multiple significant test results, including a significant main effect of nitrogen addition resulting in an increase in frequency and multiple significant interactions between burn and nitrogen addition treatments (Table 2). Analyses preformed at 97% SIL identified similar taxa with four of the nine significant responders at 93% SIL in the top ten most significant responders at 97% SIL (Table S4). However, because OTUs were broken up into more exclusive taxa, only two taxa were found to have significant responses after multiple testing corrections (FDR = 0.05). At 97% SIL, a Chloroflexi OTU had a significant interaction between burn and nitrogen with greater abundance in burn than burn+nitrogen treatments and a Gemmatimonas OTU had a significant main effect of nitrogen and with greater abundance in the presence on nitrogen enrichment (Table S4).

**Table 2:** Taxa with significant response to the addition of nitrogen at 93% SIL.

OTU #	number ofseqs in OTU	Name	Effect	Contrast	Fold Change	stderr	*q*-value
213	818	Nitrospira	nitrogen	C vs N	7.3700	1.5596	0.0343
213	818	Nitrospira	burn*nitrogen	B vs BN	11.4001	2.2035	0.0204
690	304	Acidobacteria Gp1	nitrogen	C vs N	8.8649	1.6137	0.0148
982	177	Acidobacteria Gp4	burn*nitrogen	B vs BN	−10.1861	2.1063	0.0319
2499	50	Ohtaekwangia	burn*nitrogen	C vs BN	−9.4671	1.9053	0.0267
2770	653	Acidobacteria Gp3	nitrogen	C vs N	1.2752	0.238	0.0155
2770	653	Acidobacteria Gp3	burn*nitrogen	C vs N	1.9011	0.3366	0.0126
2784	93	Gammaproteobacteria	nitrogen	C vs N	7.5369	1.41	0.0155
2784	93	Gammaproteobacteria	burn*nitrogen	B vs BN	11.7232	1.9941	0.0094
4308	75	Burkholderiales	nitrogen	C vs N	−7.4906	1.5646	0.0319
4524	88	Flavobacterium	burn*nitrogen	N vs B	−11.1137	2.409	0.0418
4697	100	Alphaproteobacteria	nitrogen	C vs N	9.6399	1.2231	0.00058
4697	100	Alphaproteobacteria	burn*nitrogen	N vs B	−10.7196	1.671	0.00586
4697	100	Alphaproteobacteria	burn*nitrogen	B vs BN	10.4106	1.7297	0.00921
4697	100	Alphaproteobacteria	burn*nitrogen	C vs UBN	8.8693	1.7297	0.0204
4697	100	Alphaproteobacteria	burn*nitrogen	C vs BN	8.5602	1.7864	0.0319

Individual taxa with significant response to treatments are shown. The specific contrast in which a significant difference is indicated (C = control, N = nitrogen, B = Burn, BN = burn+nitrogen, UBN = unburned+nitrogen). Standard error (stderr) is shown as well as false discovery rate corrected p-values (q-values).

## Discussion

An increasing number of studies are employing deep sequencing to quantify microbial communities, taking advantage of the large volume of sequences produced to determine community diversity (e.g. [21], [48], [49], [50], [51]), while others have extended this approach to allow examination of microbial community responses to a broad range of environmental perturbations [52], [53], [54], [55], [56]. The current study classified 16S rRNA sequences from soil bacteria as OTUs across long-term experimental treatments within a tallgrass prairie research site in the Central Plains (Konza Prairie LTER). We generated rarefaction curves at each %SIL to determine the best level for more detailed analyses of community diversity and taxonomic response to the treatments. We found that 93% SIL provided the greatest taxonomic resolution where we could be certain our sampling was sufficient (Figure 1). Furthermore, we tested the robustness of our analyses using the 93% SIL cutoff by also performing analyses using OTUs calculated at the more traditional 97% SIL and found that the results were highly similar. Because this study utilized the V3 region of the 16S rRNA gene, it is possible that our results provide a conservative estimate, perhaps an underestimate, of community diversity [57]. A recent study however, showed that the V3 region of the 16S performs well for sequence frequencies similar to those found in soils [33]. Although the sequences obtained here using the first generation of 454 sequencing technology were shorter and less numerous than those that can be obtained with the current technology, we were able to investigate the effect of replicated plot-level perturbation on both community diversi1ty and individual taxa responses. Within this system, we set out to address three hypotheses:

1) Soil bacterial community composition is altered in response to nutrient enrichment and/or contrasting fire regimes. We found that nitrogen amendment significantly reduced community richness and diversity, but the other treatments (phosphorus addition, contrasting fire regimes) had no effect (Figure 3). The lack of response to phosphorus additions is not surprising, and may be due to the relatively low levels of phosphorus added and to the high capacity of these calcium-rich soils to bind phosphorus. This is also consistent with the lack of response in plant productivity or species composition to added phosphorus in this experiment (*unpublished data*). The lack of bacterial community response to fire treatments was somewhat surprising, as previous studies have reported significant differences in plant community composition [23], belowground plant biomass [26], and soil carbon and nitrogen pools and fluxes [18], [25], [58] in annually burned and unburned tallgrass prairie. These results suggest that soil bacterial communities may not be affected as strongly as plant communities to changes in the soil environment associated with burning. In addition, the contrast in responses of plant and bacterial communities to fire treatments is consistent with other studies [10], and reinforces the suggestion that different factors may structure plant and microbial responses to the same treatments [59].

The decrease in taxonomic richness caused by nitrogen addition could result from consistent loss of particular taxa or an increase in dominant taxa and concurrent loss of multiple rare taxa. Although we were able to identify individual taxa with significant increases and decreases in abundance in other analyses, the number of responsive taxa was insufficient to explain the apparent loss in taxonomic richness observed. Interestingly, we also found a significant increase in Simpson’s dominance in response to nitrogen enrichment. Furthermore, we found that there was a significant reduction of extremely rare taxa in response to nitrogen addition. Taken together, our data suggest that an increase in species dominance, decrease in rare taxa and decrease in overall species richness result from nitrogen amendment of tallgrass prairie soils. Reduction in diversity in fertilized plots was also observed in arctic tundra [60] suggesting that nitrogen enrichment may reduce niche space and therefore community diversity. In contrast no reduction in diversity was observed in response to nitrogen enrichment in another grassland ecosystem (Cedar Creek), indicating that species richness responses to nitrogen enrichment may be site or soil specific [10].

2) Soil microbial community structure is altered in response to nutrient enrichment and/or contrasting fire regimes. We used a phylogenetic approach (UniFrac) to test this hypothesis. We found that the nitrogen enrichment treatment resulted in distinct bacterial communities as compared to control plots, whereas the burn treatments did not (Figure 3). This is somewhat surprising given that previous studies have shown significant biogeochemical changes in the soil in response to different fire frequencies [15], [18], [25], [28]. One interpretation is that there are threshold responses in microbial community changes to enhanced soil nitrogen availability, and the changes associated with different fire treatments are not sufficient to elicit the responses observed to added nitrogen. The community structure responses we report in response to nitrogen enrichment are similar to that observed in a study of multiple terrestrial ecosystems where it was shown that nitrogen enrichment significantly shifted bacterial communities [12] suggesting that this may be a more general effect of nitrogen enrichment. When we tested for treatment elicited alterations in edaphic properties we found that nitrogen addition increased extractable inorganic nitrogen and nitrate concentrations by 0.92-fold and 6.9-fold, respectively, while the annual burn treatment reduced extractable soil inorganic nitrogen by 0.13-fold, and concurrent annual burning and nitrogen treatments resulted in no difference from control. These data are consistent with previous studies documenting lower inorganic nitrogen concentrations and lower net nitrogen mineralization rates in annually burned compared to unburned prairie, which results from the greater nitrogen limitation/immobilization that accompanies annual burning [18], [25]. While these are long-term experiments with ongoing treatments conducted as part of the Konza Prairie LTER program, seasonal dynamics in both soil properties and in microbial communities have been documented [28], [58] making it difficult to know how the measured soil properties directly relate to the measurements of the microbial community presented. In addition, we should point out the soil property data were not measured on the same samples from which DNA was extracted, but on alternate samples collected prior to those used for DNA extraction, thus correlative analyses were not appropriate. Subsequent studies with concurrent measurements of soil microbial communities and soil properties over time and on the same samples will be needed to more conclusively test for relationships between soil properties and microbial community structure.

3) Individual taxa frequencies are altered in response to nutrient enrichment and/or contrasting fire regimes. We found that individual bacterial taxa frequencies at 93% SIL were altered in response to nitrogen treatment and the interaction of nitrogen and burn treatments (Table 2) but bacterial phylum-level taxa frequencies were not significantly altered in response to either nitrogen or burn treatments. Interestingly, the taxa with frequencies significantly altered in response to treatments at 93% SIL were constituents of the phylum showing the largest, however non-significant changes in response to treatments (Figure S3). It seems likely that at the resolution of bacterial phylum, the signal of individual taxa responses was masked because members of the phylum were responding differently, or that responsive members may have made up a minority of the constituents of the phylum. It is also possible that the significant shifts in individual taxa may be indicative of the active community of bacteria present, but that at the phylum level the dormant community of bacteria present in the soil masks these responses. Differentiating these two possibilities and others will be needed to understand why different taxonomic resolutions show different responsive taxa (Tables 2, S4) but do not show differences in community diversity and structure (Figures 3, 4, S3, S4, S5). Additionally, in all treatments the same five bacterial phylum were the most abundant: 1) Proteobacteria (40%) 2) Verrucomicrobia (11.7%), 3) Bacteriodetes (10.1%), 4) Acidobacteria (8.1%), 5) Firmicutes (5.7%) and 6) Actinobacteria (4.5%) with the remaining bacterial phylum combining to 20% (Figure 2) and these taxa have been previously found to be highly abundant in soils [61], [62]. When we tested for individual taxon responses at 93% SIL we found that treatment interactions accounted for the majority of the significant effects, suggesting that the responses of bacterial taxa are context dependent. This is interesting because we found an OTU identified as Nitrospira increased in abundance in response to the addition of nitrogen fertilizer but only in the presence of the burn treatment (Table 2). In a recent study of nitrogen enrichment in two distinct study sites, Ramirez *et al.* [22] found reduced abundance of Nitrospira in response to increasing levels of nitrogen enrichment in an agricultural field at the Kellogg Biological Station. Together our data and that of Ramirez *et al.* [22] suggest that the responses of Nitrospira taxa to nitrogen enrichment may be highly context dependent, with a decrease in response to nitrogen alone in at least some ecosystems, but an increase in response to nitrogen with concurrent burn treatment. However, this raises questions about the generality of other bacterial species treatment responses and whether simplistic models of increases/decreases in response to a particular treatment are accurate. Furthermore, we found Gammaproteobacteria increased in response to nitrogen addition in the presence of the burn treatment (Table 2), which is consistent with a reported increase in Gammaproteobacteria in response to nitrogen enrichment in three other sites (grasslands at Cedar Creek, an agricultural field at the Kellogg Biological Station, arctic tundra [22], [60]). This suggests that for at least Gammaproteobacteria taxa, the observed response may not be dependent on the presence of the burn treatment like that of Nitrospira taxa. More studies of bacterial taxa responses to treatment combinations are required before we know if the context dependant nature of Nitrospira responses are representative of bacterial taxa in general, or if the less context-dependent responses of bacteria like Gammaproteobacteria taxa are more typical.

Our results show that chronic soil nitrogen enrichment can significantly alter bacterial community diversity, structure, and individual taxa abundance, which has important implications for both managed and natural grassland ecosystems. Chronic nitrogen enrichment significantly impacted bacterial community diversity and community structure, with individual groups such as OTUs identified as Nitrospira and Gammaproteobacteria exhibiting changes in relative abundance under long-term nitrogen enrichment. Surprisingly, we found little response to burning by the soil microbial community, although contrasting fire regimes result in significant changes in the soil environment and in plant productivity and plant community composition in these grasslands. These results suggest that the factors controlling plant and microbial community responses to environmental change may differ, and that changes in plant communities may not correlate with changes in soil bacterial communities. Additional studies will be needed to more fully understand the factors underlying observed microbial community responses to nitrogen enrichment, and the consequences of changes in microbial community structure for ecosystem function.

## Supporting Information

Figure S1 **Experimental design for the Belowground Plot Experiment.** Treatments are abbreviated: N = nitrogen addition, C = no nitrogen addition, P = phosphorous addition, NP = nitrogen and phosphorus addition; red outline = annually burned plots; shaded areas = treatments not sampled.
(TIFF)

Figure S2 **Rarefaction curves calculated independently for each plot.** Rarefaction curves for A) 93% SIL and B) 97% SIL are shown.
(TIFF)

Figure S3 **Proportion of taxa represented in each phylum.** The mean proportional abundance of each bacterial phylum identified is shown for control and treatments (nitrogen, burn and burn+nitrogen) as well as for the mean for all plots. In each case there was no significant effect of treatment on phylum proportional abundance.
(TIFF)

Figure S4 **Mean alpha diversity at 97% SIL.** Treatments included were control (black), nitrogen amended (dark gray), burned (light gray), and burn+nitrogen treatment (white). A) Fisher’s alpha, B) Taxonomic richness, C) Simpson’s dominance, D) number of singlets (extremely rare taxa), E) Shannon diversity and F) taxonomic evenness are shown. An * indicates significant at p<0.05 in mixed model ANOVA. Error bars indicate standard errors.
(TIFF)

Figure S5 **UniFrac Principle Coordinate Analysis plot at 97% SIL.** Treatments indicated are control (open black), nitrogen addition (filled black), burned (open grey), and nitrogen+burn (filled grey) treatments were plotted. Clustering based on nitrogen treatment is outlined (nitrogen in solid line and no nitrogen addition in dashed line).
(TIFF)

Table S1 **Primers for barcoded deep sequencing.** The primers were produced by adding unique barcode sequences (underlined) between the “A” sequencing primer of Margulies *et al.* [34] and the reverse 16S primer U529R (**bold**) of Watanabe *et al.* [32]. As sequencing was done in only the reverse direction, no barcode was necessary within the “B” construct.
(DOCX)

Table S2 **Summary of read count per plot before and after quality filtering.** Counts for the raw number of reads (Raw) generated per plot as well as the number that were retained after quality filtering (Final) are shown.
(DOCX)

Table S3 **Table of all phylum found in Belowground Plot Experiment samples.** Mean frequency in percentages for each phylum for each treatment is indicated.
(DOCX)

Table S4 **Taxa with significant response to the addition of nitrogen at 97% SIL.** The specific contrast for which a significant difference was observed is indicated (C = control, N = nitrogen, B = Burn, BN = burn+nitrogen). Standard error (stderr) is shown as well as false discovery rate corrected p-values (q-values).
(DOCX)
