# Supplementary figures and images for: Long-Term Nitrogen Amendment Alters the Diversity and Assemblage of Soil Bacterial Communities in Tallgrass Prairie
Source: PLoS One. 2013 Jun 28;8(6):e67884. doi: 10.1371/journal.pone.0067884 (PMC3695917; doi:10.1371/journal.pone.0067884)

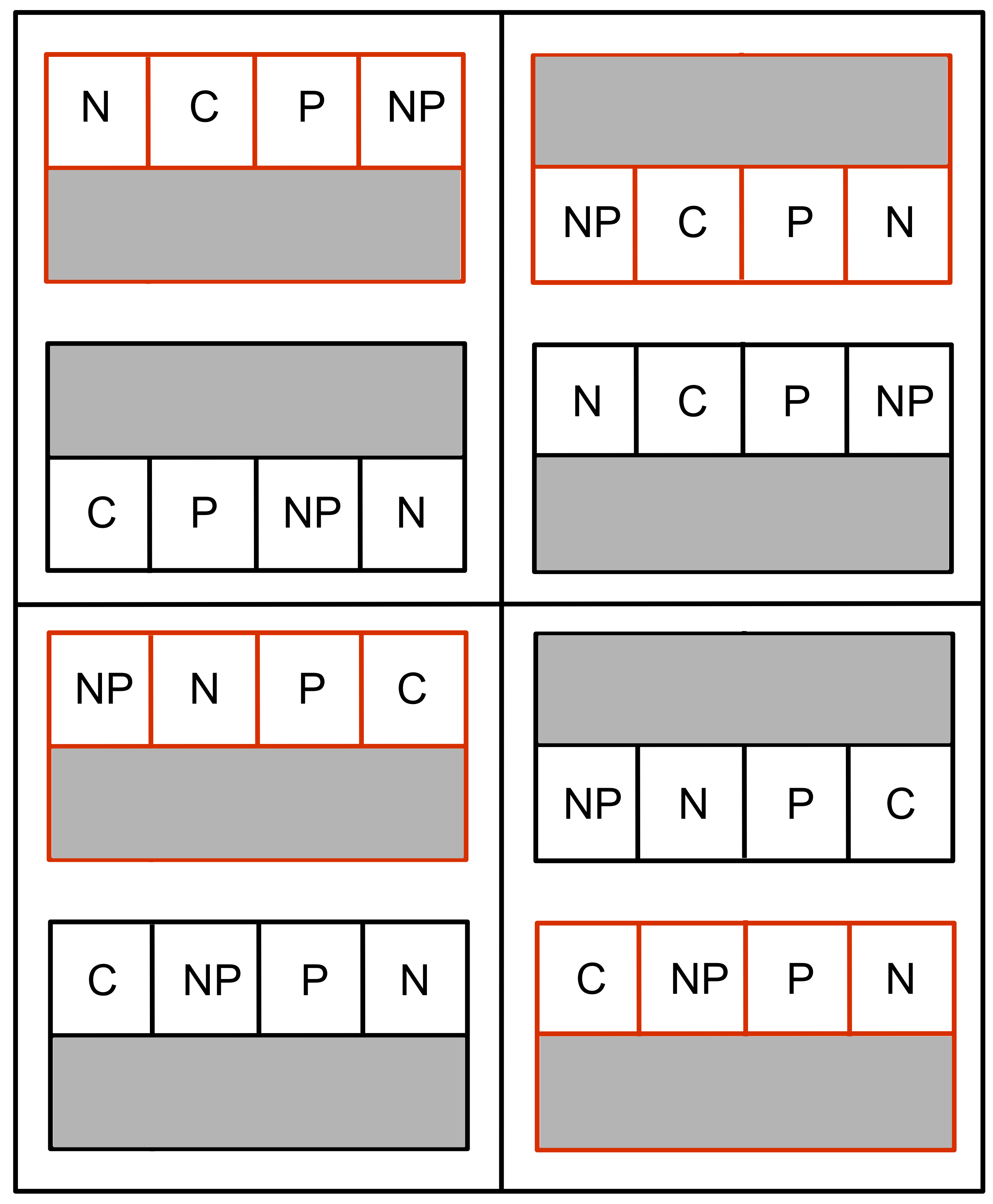

Supplement: Figure S1 — Experimental design for the Belowground Plot Experiment. Treatments are abbreviated: N = nitrogen addition, C = no nitrogen addition, P = phosphorous addition, NP = nitrogen and phosphorus addition; red outline = annually burned plots; shaded areas = treatments not sampled. (TIFF) [file pone.0067884.s001.tif]

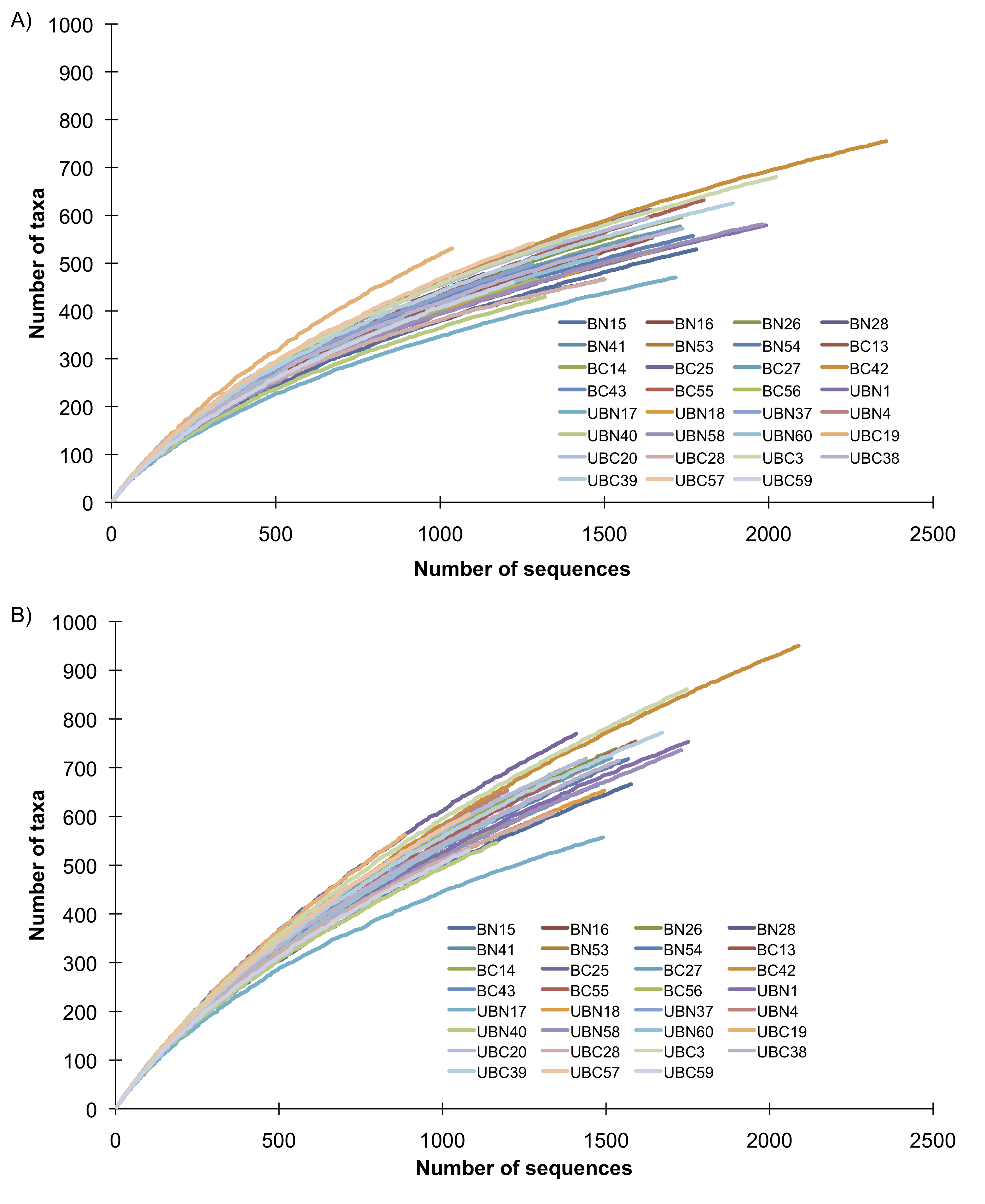

Supplement: Figure S2 — Rarefaction curves calculated independently for each plot. Rarefaction curves for A) 93% SIL and B) 97% SIL are shown. (TIFF) [file pone.0067884.s002.tif]

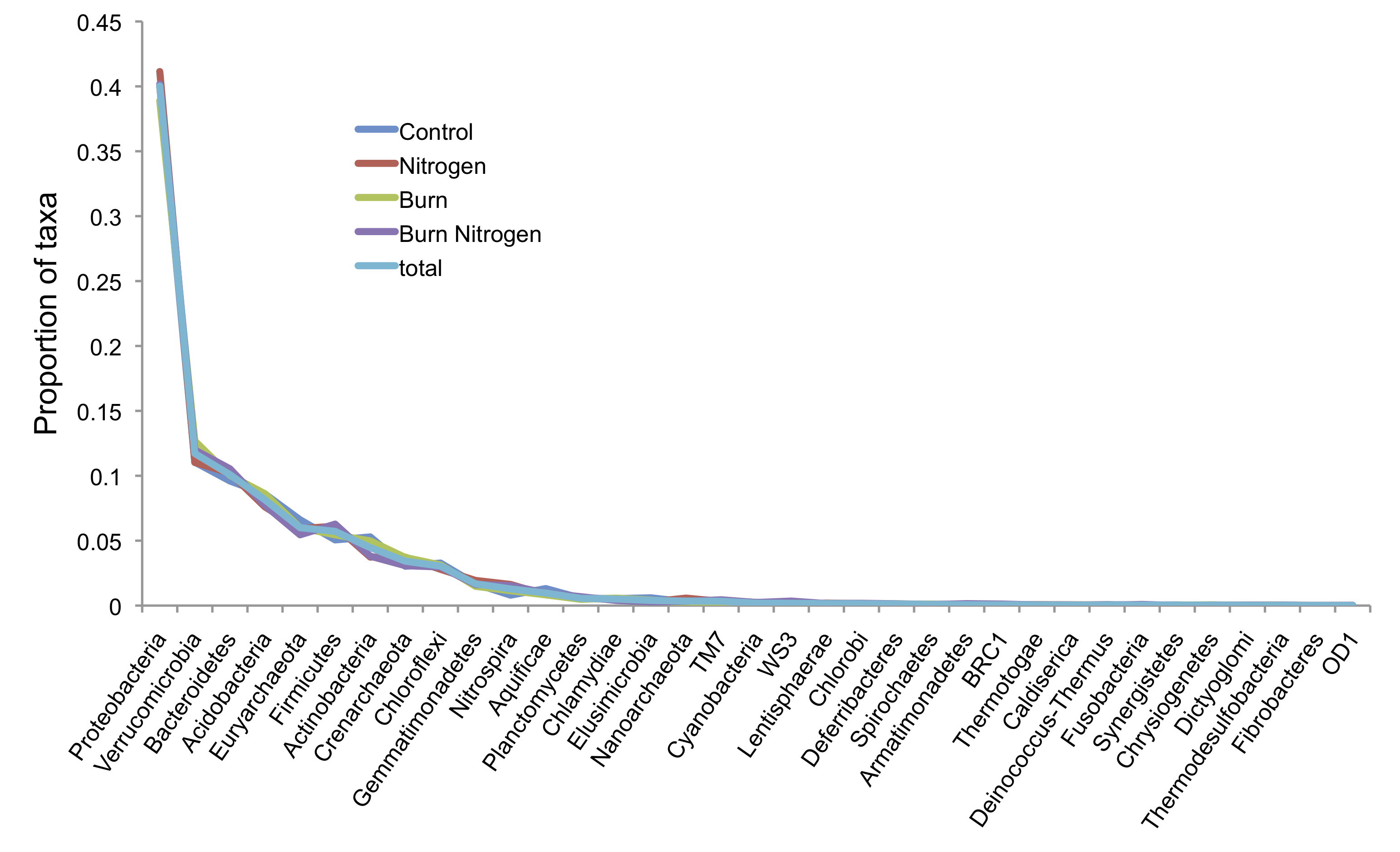

Supplement: Figure S3 — Proportion of taxa represented in each phylum. The mean proportional abundance of each bacterial phylum identified is shown for control and treatments (nitrogen, burn and burn+nitrogen) as well as for the mean for all plots. In each case there was no significant effect of treatment on phylum proportional abundance. (TIFF) [file pone.0067884.s003.tif]

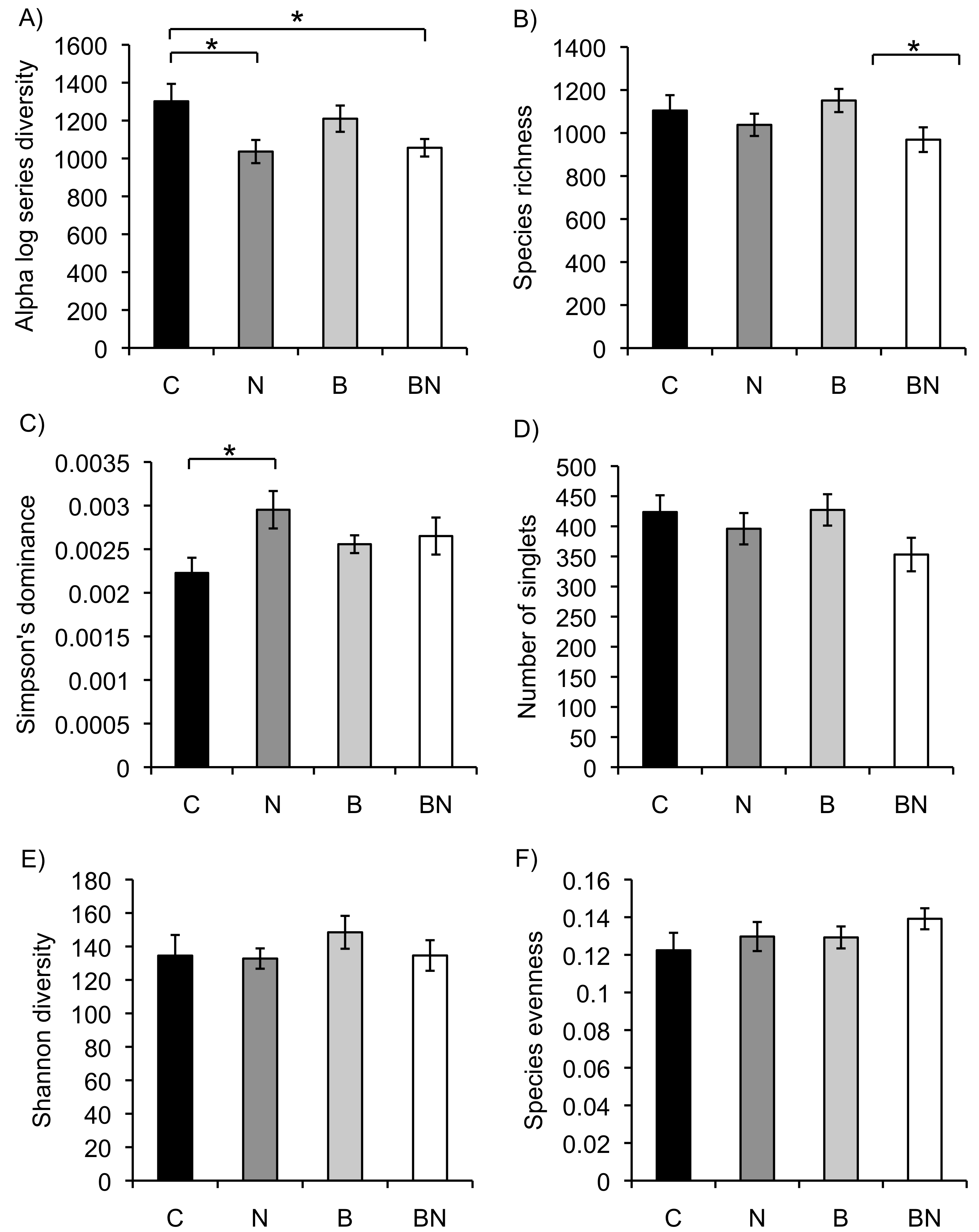

Supplement: Figure S4 — Mean alpha diversity at 97% SIL. Treatments included were control (black), nitrogen amended (dark gray), burned (light gray), and burn+nitrogen treatment (white). A) Fisher’s alpha, B) Taxonomic richness, C) Simpson’s dominance, D) number of singlets (extremely rare taxa), E) Shannon diversity and F) taxonomic evenness are shown. An * indicates significant at p<0.05 in mixed model ANOVA. Error bars indicate standard errors. (TIFF) [file pone.0067884.s004.tif]

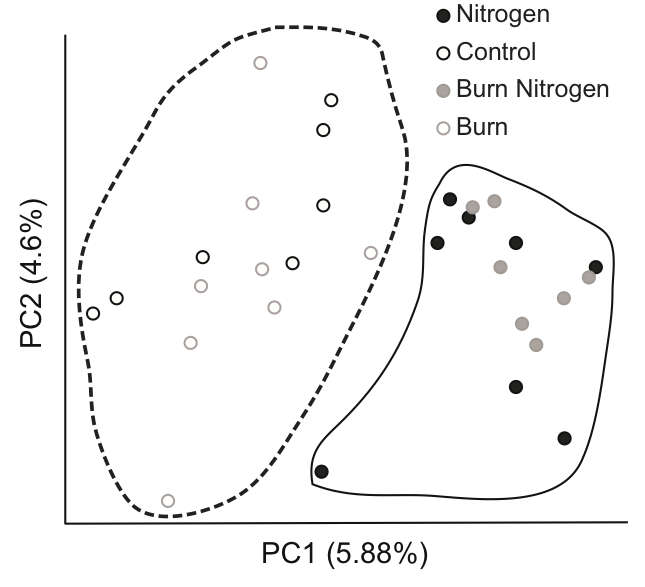

Supplement: Figure S5 — UniFrac Principle Coordinate Analysis plot at 97% SIL. Treatments indicated are control (open black), nitrogen addition (filled black), burned (open grey), and nitrogen+burn (filled grey) treatments were plotted. Clustering based on nitrogen treatment is outlined (nitrogen in solid line and no nitrogen addition in dashed line). (TIFF) [file pone.0067884.s005.tif]
