# Supplementary material for: Long-Term Nitrogen Amendment Alters the Diversity and Assemblage of Soil Bacterial Communities in Tallgrass Prairie
Source: PLoS One. 2013 Jun 28;8(6):e67884. doi: 10.1371/journal.pone.0067884 (PMC3695917; doi:10.1371/journal.pone.0067884)
Supplement: Table S1 — Primers for barcoded deep sequencing. The primers were produced by adding unique barcode sequences (underlined) between the “A” sequencing primer of Margulies et al. [34] and the reverse 16S primer U529R (bold) of Watanabe et al. [32]. As sequencing was done in only the reverse direction, no barcode was necessary within the “B” construct. (DOCX) [file pone.0067884.s006.docx]

Table S1. Primers for barcoded deep sequencing.

| **Primer** | **Sequence** |
| --- | --- |
| U341F-FC-B | GCCTTGCCAGCCCGCTCAG**CCTACGGGRSGCAGCAG** |
|  |  |
| U529R-FC-A3 | GCCTCCCTCGCGCCATCAGACTCA**ACCGCGGCKGCTGGC** |
| U529R-FC-A9 | GCCTCCCTCGCGCCATCAGAGCAG**ACCGCGGCKGCTGGC** |
| U529R-FC-A11 | GCCTCCCTCGCGCCATCAGTATCA**ACCGCGGCKGCTGGC** |
| U529R-FC-A14 | GCCTCCCTCGCGCCATCAGAGTAT**ACCGCGGCKGCTGGC** |
| U529R-FC-A16 | GCCTCCCTCGCGCCATCAGCTACG**ACCGCGGCKGCTGGC** |
| U529R-FC-A18 | GCCTCCCTCGCGCCATCAGACTAG**ACCGCGGCKGCTGGC** |
| U529R-FC-A20 | GCCTCCCTCGCGCCATCAGTCTCT**ACCGCGGCKGCTGGC** |
| U529R-FC-A22 | GCCTCCCTCGCGCCATCAGACTCG**ACCGCGGCKGCTGGC** |
| U529R-FC-A24 | GCCTCCCTCGCGCCATCAGACTCT**ACCGCGGCKGCTGGC** |
| U529R-FC-A25 | GCCTCCCTCGCGCCATCAGTGTCA**ACCGCGGCKGCTGGC** |
| U529R-FC-A27 | GCCTCCCTCGCGCCATCAGCTACT**ACCGCGGCKGCTGGC** |
| U529R-FC-A29 | GCCTCCCTCGCGCCATCAGAGCTG**ACCGCGGCKGCTGGC** |
| U529R-FC-A33 | GCCTCCCTCGCGCCATCAGTGATG**ACCGCGGCKGCTGGC** |
| U529R-FC-A35 | GCCTCCCTCGCGCCATCAGAGCGC**ACCGCGGCKGCTGGC** |
| U529R-FC-A40 | GCCTCCCTCGCGCCATCAGTCACT**ACCGCGGCKGCTGGC** |
| U529R-FC-A42 | GCCTCCCTCGCGCCATCAGTGTGC**ACCGCGGCKGCTGGC** |
| U529R-FC-A46 | GCCTCCCTCGCGCCATCAGTACTA**ACCGCGGCKGCTGGC** |
| U529R-FC-A49 | GCCTCCCTCGCGCCATCAGAGATG**ACCGCGGCKGCTGGC** |
| U529R-FC-A53 | GCCTCCCTCGCGCCATCAGCGATG**ACCGCGGCKGCTGGC** |
| U529R-FC-A57 | GCCTCCCTCGCGCCATCAGCTAGT**ACCGCGGCKGCTGGC** |
| U529R-FC-A64 | GCCTCCCTCGCGCCATCAGTACGT**ACCGCGGCKGCTGGC** |
| U529R-FC-A66 | GCCTCCCTCGCGCCATCAGTGTAG**ACCGCGGCKGCTGGC** |
| U529R-FC-A75 | GCCTCCCTCGCGCCATCAGATATG**ACCGCGGCKGCTGGC** |
| U529R-FC-A76 | GCCTCCCTCGCGCCATCAGTCACA**ACCGCGGCKGCTGGC** |
| U529R-FC-A77 | GCCTCCCTCGCGCCATCAGTCTGA**ACCGCGGCKGCTGGC** |
| U529R-FC-A86 | GCCTCCCTCGCGCCATCAGTACAG**ACCGCGGCKGCTGGC** |
| U529R-FC-A88 | GCCTCCCTCGCGCCATCAGTACGA**ACCGCGGCKGCTGGC** |
| U529R-FC-A89 | GCCTCCCTCGCGCCATCAGTGTAC**ACCGCGGCKGCTGGC** |
| U529R-FC-A90 | GCCTCCCTCGCGCCATCAGATACG**ACCGCGGCKGCTGGC** |
| U529R-FC-A94 | GCCTCCCTCGCGCCATCAGACTGA**ACCGCGGCKGCTGGC** |
| U529R-FC-A95 | GCCTCCCTCGCGCCATCAGTGTGT**ACCGCGGCKGCTGGC** |
| U529R-FC-A96 | GCCTCCCTCGCGCCATCAGATCTG**ACCGCGGCKGCTGGC** |
|  |  |
