# Supplementary material for: Long-Term Nitrogen Amendment Alters the Diversity and Assemblage of Soil Bacterial Communities in Tallgrass Prairie
Source: PLoS One. 2013 Jun 28;8(6):e67884. doi: 10.1371/journal.pone.0067884 (PMC3695917; doi:10.1371/journal.pone.0067884)
Supplement: Table S2 — Summary of read count per plot before and after quality filtering. Counts for the raw number of reads (Raw) generated per plot as well as the number that were retained after quality filtering (Final) are shown. (DOCX) [file pone.0067884.s007.docx]

Table S2: Summary of read count per plot before and after quality filtering

| Plot | Raw | Final |
| --- | --- | --- |
| 1 | 2891 | 1993 |
| 3 | 2959 | 2023 |
| 4 | 2155 | 1394 |
| 13 | 2775 | 1645 |
| 14 | 2257 | 1603 |
| 15 | 2716 | 1781 |
| 16 | 1788 | 1138 |
| 17 | 2819 | 1718 |
| 18 | 2807 | 1677 |
| 19 | 1651 | 1038 |
| 20 | 2517 | 1633 |
| 25 | 2508 | 1642 |
| 26 | 2476 | 1736 |
| 27 | 2496 | 1731 |
| 28 | 1993 | 1502 |
| 37 | 2578 | 1282 |
| 38 | 2420 | 1739 |
| 39 | 2619 | 1892 |
| 40 | 2078 | 1320 |
| 41 | 1754 | 980 |
| 42 | 3483 | 2359 |
| 43 | 2416 | 1668 |
| 53 | 1763 | 1180 |
| 54 | 2420 | 1770 |
| 55 | 2489 | 1803 |
| 56 | 2024 | 1294 |
| 57 | 2116 | 1283 |
| 58 | 2979 | 1983 |
| 59 | 1824 | 1224 |
| 60 | 1998 | 1477 |
