# Supplementary material for: Long-Term Nitrogen Amendment Alters the Diversity and Assemblage of Soil Bacterial Communities in Tallgrass Prairie
Source: PLoS One. 2013 Jun 28;8(6):e67884. doi: 10.1371/journal.pone.0067884 (PMC3695917; doi:10.1371/journal.pone.0067884)
Supplement: Table S3 — Table of all phylum found in Belowground Plot Experiment samples. Mean frequency in percentages for each phylum for each treatment is indicated. (DOCX) [file pone.0067884.s008.docx]

Table S3: Table of all phylum found in Belowground Plot Experiment samples.

| **Phylum** | **Control** | **Nitrogen** | **Burn** | **Burn Nitrogen** | **Total** |
| --- | --- | --- | --- | --- | --- |
| Acidobacteria | 8.59 | 7.60 | 8.61 | 7.71 | 8.14 |
| Actinobacteria | 5.29 | 3.74 | 4.98 | 3.81 | 4.47 |
| Aquificae | 1.31 | 0.83 | 0.83 | 0.97 | 0.97 |
| Armatimonadetes | 0.06 | 0.02 | 0.07 | 0.15 | 0.07 |
| Bacteroidetes | 9.60 | 10.21 | 10.03 | 10.54 | 10.09 |
| BRC1 | 0.06 | 0.03 | 0.04 | 0.12 | 0.06 |
| Caldiserica | 0.03 | 0.06 | 0.04 | 0.05 | 0.05 |
| Chlamydiae | 0.54 | 0.49 | 0.57 | 0.40 | 0.51 |
| Chlorobi | 0.13 | 0.15 | 0.11 | 0.18 | 0.14 |
| Chloroflexi | 3.28 | 2.80 | 3.13 | 2.97 | 3.04 |
| Chrysiogenetes | 0.01 | 0.01 | 0.06 | 0.03 | 0.03 |
| Crenarchaeota | 3.03 | 3.72 | 3.70 | 3.07 | 3.42 |
| Cyanobacteria/Chloroplast | 0.25 | 0.09 | 0.19 | 0.24 | 0.19 |
| Deferribacteres | 0.11 | 0.10 | 0.13 | 0.14 | 0.12 |
| Deinococcus-Thermus | 0.07 | 0.05 | 0.01 | 0.03 | 0.04 |
| Dictyoglomi | 0.03 | 0.04 | 0.01 | 0.03 | 0.03 |
| Elusimicrobia | 0.60 | 0.29 | 0.42 | 0.29 | 0.40 |
| Euryarchaeota | 6.61 | 5.92 | 5.99 | 5.45 | 6.00 |
| Fibrobacteres | 0.00 | 0.02 | 0.01 | 0.02 | 0.01 |
| Firmicutes | 5.06 | 6.12 | 5.45 | 6.28 | 5.72 |
| Fusobacteria | 0.04 | 0.02 | 0.02 | 0.09 | 0.04 |
| Gemmatimonadetes | 1.67 | 1.94 | 1.48 | 1.63 | 1.68 |
| Lentisphaerae | 0.14 | 0.19 | 0.15 | 0.16 | 0.16 |
| Nanoarchaeota | 0.29 | 0.58 | 0.20 | 0.33 | 0.35 |
| Nitrospira | 0.80 | 1.63 | 1.14 | 1.54 | 1.28 |
| OD1 | 0.00 | 0.02 | 0.00 | 0.02 | 0.01 |
| Planctomycetes | 0.57 | 0.54 | 0.48 | 0.68 | 0.56 |
| Proteobacteria | 40.01 | 41.14 | 38.89 | 40.17 | 40.02 |
| Spirochaetes | 0.11 | 0.05 | 0.10 | 0.08 | 0.08 |
| Synergistetes | 0.06 | 0.04 | 0.04 | 0.01 | 0.04 |
| Thermodesulfobacteria | 0.05 | 0.02 | 0.01 | 0.00 | 0.02 |
| Thermotogae | 0.06 | 0.07 | 0.03 | 0.05 | 0.05 |
| TM7 | 0.35 | 0.32 | 0.27 | 0.45 | 0.34 |
| Verrucomicrobia | 11.05 | 11.02 | 12.68 | 11.99 | 11.71 |
| WS3 | 0.16 | 0.14 | 0.09 | 0.35 | 0.17 |
