# Supplementary material for: Long-Term Nitrogen Amendment Alters the Diversity and Assemblage of Soil Bacterial Communities in Tallgrass Prairie
Source: PLoS One. 2013 Jun 28;8(6):e67884. doi: 10.1371/journal.pone.0067884 (PMC3695917; doi:10.1371/journal.pone.0067884)
Supplement: Table S4 — Taxa with significant response to the addition of nitrogen at 97% SIL. The specific contrast for which a significant difference was observed is indicated (C = control, N = nitrogen, B = Burn, BN = burn+nitrogen). Standard error (stderr) is shown as well as false discovery rate corrected p-values (q-values). (DOCX) [file pone.0067884.s009.docx]

Table S4: Taxa with significant response to the addition of nitrogen at 97%SIL

| **OTU #** | **Number of seqs in OTU** | **Name** | **Effect** | **Contrast** | **Fold Change** | **stderr** | ***p*-value** | ***q-value*** |
| --- | --- | --- | --- | --- | --- | --- | --- | --- |
| 5001 | 53 | Chloroflexi | burn*nitrogen | B vs BN | 9.5817 | 1.4351 | 1.68868E-06 | 0.04160000 |
| 2081 | 155 | Gemmatimonas | nitrogen | C vs N | 8.8824 | 1.3638 | 2.38941E-06 | 0.04160000 |
| 5001 | 53 | Chloroflexi | burn*nitrogen | C vs BN | 9.5195 | 1.4847 | 2.96646E-06 | 0.04160000 |
| 1951 | 32 | Acidobacteria Gp1 | nitrogen | C vs N | 8.2223 | 1.3840 | 8.25137E-06 | 0.07424247 |
| 3601 | 10 | Cyanobacteria | burn*nitrogen | B vs BN | 7.9029 | 1.3388 | 8.96746E-06 | 0.07424247 |
| 3601 | 10 | Cyanobacteria | burn*nitrogen | N vs BN | 7.9029 | 1.3559 | 1.05743E-05 | 0.07424247 |
| 5001 | 53 | Chloroflexi | burn*nitrogen | N vs BN | 8.2415 | 1.4351 | 1.27895E-05 | 0.07696753 |
| 3601 | 10 | Cyanobacteria | burn*nitrogen | C vs BN | 7.9029 | 1.3997 | 1.58718E-05 | 0.08357727 |
| 2130 | 161 | Acidobacteria Gp6 | nitrogen | C vs N | -7.4739 | 1.3609 | 2.24326E-05 | 0.10499995 |
| 2081 | 155 | Gemmatimonas | burn*nitrogen | B vs BN | 10.4667 | 1.9273 | 2.57408E-05 | 0.10843614 |
| 5001 | 53 | Chloroflexi | nitrogen | C vs N | 5.4298 | 1.0152 | 3.10054E-05 | 0.11873990 |
| 135 | 703 | Nitrospira | burn*nitrogen | B vs BN | 11.3681 | 2.1697 | 3.97431E-05 | 0.13951873 |
| 2081 | 155 | Gemmatimonas | burn*nitrogen | C vs BN | 10.3709 | 1.9971 | 4.41818E-05 | 0.14317001 |
| 2130 | 161 | Acidobacteria Gp6 | burn*nitrogen | C vs N | -9.7500 | 1.9242 | 5.89157E-05 | 0.17727807 |
| 2258 | 45 | Verrucomicrobia | burn*nitrogen | N vs B | 8.3665 | 1.6653 | 6.50503E-05 | 0.18268801 |
| 5494 | 28 | Acidobacteria Gp4 | burn*nitrogen | B vs BN | -6.6806 | 1.3610 | 8.48425E-05 | 0.21522956 |
| 6111 | 452 | Acidobacteria Gp1 | burn*nitrogen | C vs N | 10.8316 | 2.2113 | 8.68558E-05 | 0.21522956 |
| 2258 | 45 | Verrucomicrobia | burn*nitrogen | B vs BN | -8.4012 | 1.7252 | 9.28133E-05 | 0.21721495 |
| 2698 | 25 | Ohtaekwangia | nitrogen | C vs N | -6.7607 | 1.4235 | 0.000122595 | 0.21721495 |
| 135 | 703 | Nitrospira | nitrogen | C vs N | 7.2440 | 1.5357 | 0.000132050 | 0.21721495 |
